# Effects of different manganese sources on nutrient digestibility, fecal bacterial community, and mineral excretion of weaning dairy calves

**DOI:** 10.3389/fmicb.2023.1163468

**Published:** 2023-05-18

**Authors:** Huimin Ji, Dejin Tan, Yuhua Chen, Zhiqiang Cheng, Jingwen Zhao, Miao Lin

**Affiliations:** ^1^Institute of Animal Culture Collection and Application, College of Animal Science and Technology, Yangzhou University, Yangzhou, China; ^2^Institutes of Agricultural Science and Technology Development, Yangzhou University, Yangzhou, China; ^3^Joint International Research Laboratory of Agriculture and Agri-Product Safety, The Ministry of Education of China, Yangzhou University, Yangzhou, China

**Keywords:** Holstein heifers weaning, digestibility, bacteria, mineral element, feces

## Abstract

**Introduction:**

Mn, which is an essential trace mineral for all animals, has functions in skeletal system development, carbohydrate and lipid metabolism. The aim of this study was to clarify the effects of different manganese (Mn) sources in basal diets on nutrient apparent digestibility, fecal microbes, and mineral elements excretion before and after weaning.

**Methods:**

A total of 15 Holstein heifer calves (6-week-old, 82.71 ± 1.35, mean ± standard error) were randomly designed into three groups (five each): no extra Mn supplemented (CON), 20 mg Mn/kg (dry matter basis) in the form of chelates of lysine and glutamic acid in a mixture of 1:1 (LGM), and 20 mg Mn/kg (dry matter basis) in the form of MnSO_4_. All calves were weaned at 8 weeks of age. The experiment lasted for 28 days (14 days before weaning and 14 days after weaning). Dry matter intake (DMI) was recorded daily. The animals were weighed by electronic walk-over, and body size indices were collected using tape on days −14, −1, and 14 of weaning. The feces of calves was collected to measure the apparent digestibility of nutrients (acid insoluble ash was an internal marker) and bacterial community on days −1, 1, 3, 7, and 14 of weaning. Fecal mineral concentration was determined by inductively coupled plasma emission spectroscopy on days −1, 1, 7, and 14 of weaning.

**Results:**

The results showed that, compared with the CON group, adding LGM to diets containing 158.82 mg/kg Mn increased the apparent digestibility (*P* < 0.05). The Chao 1 and Shannon index of fecal bacteria decreased at day 1 in the LGM and MnSO_4_ groups and increased after weaning. The PCoA results indicated that the LGM group was distinctly separate from the CON and MnSO_4_ groups during the whole experimental period. Significant differences (*P* < 0.05) were observed in the relative abundance of two phyla (*Proteobacteria* and *Spirochaetota*) and eight genera (*Alloprevotella, Prevotellaceae*_UCG-001, *Clostridia* UCG 014, RF39, UCG-010, *Pseudomonas, Ralstonia*, and *Treponema*) in three groups. Moreover, the LGM group showed less excretion of Fe, P, and Mn than the MnSO_4_ group.

**Discussion:**

In summary, 20 mg Mn/kg diet supplementation improved nutrient digestibility, changed the fecal microbial community, and reduced mineral excretion. Organic Mn supplementation in the diet had more advantages over the sulfate forms in weaning calves.

## 1. Introduction

Effective feeding and management of calves are linked to the future productive performance of the dairy herd on intensive farms. Therefore, the healthy development of calves, along with good growth performance, is strongly associated with the future development and economic efficiency of the farm. The rumen of a newborn calf is more similar to the stomach of monogastric animals and starts developing after receiving a solid diet (Baldwin et al., 2004). Therefore, before the rumen is fully developed, the feed digestion in calves is more dependent on enzymatic digestion in the hindgut (Guilloteau et al., 2009). Gut microbial colonization occurs from the day of birth and stimulates the development of animal defense mechanisms (Yáñez-Ruiz et al., 2015). The gut microbiota community in early life has long-term impacts on host health (Malmuthuge and Guan, 2017), and different positions of the GIT contain various communities (Malmuthuge et al., 2014), i.e., microbial diversity and richness increase from the proximal small intestine to the distal colon (Malmuthuge et al., 2015). The strong gut microbiota can form a barrier of bacterial membrane on the surface of intestinal epithelial cells, protect the host from harmful foreign bacteria, and inhibit the invasion and reproduction of gut pathogens by competing for nutrients (Bibiloni et al., 2005). Additionally, microbial diversity is also influenced by several factors, such as age, diet, and intake (Guilloteau et al., 2009), and trace mineral source (i.e., solubility) is one of the known influencing factors (Faulkner et al., 2017).

Spears (1996) believed that the mineral form is more important than the quantity. Chelating minerals can produce stable soluble molecules with high bioavailability and are better absorbed than inorganic forms, due to the fact that chelated minerals are metal ions bound to organic substances such as amino acids, peptides, or polysaccharides and are then absorbed through the pathway of ion-bound organic ligands, avoiding their interaction with other molecules (Kratzer and Vohra, 1996). Manganese (Mn), an essential trace mineral for all animals, is absorbed in the GIT after ingestion and then transported to mitochondria-containing organs such as the liver, pancreas, and pituitary, where it is rapidly enriched (Deng et al., 2013). Mn has functions in skeletal system development, carbohydrate and lipid metabolism, and the innate immune response (Santamaria, 2008; Haase, 2018). Organic sources (glycine amino acid-chelated zinc, manganese, and copper) presented higher bioavailability compared with inorganic sources (zinc sulfate monohydrate, manganese sulfate monohydrate, and copper sulfate pentahydrate) in Murrah buffalo (Mudgal et al., 2019). In addition, previous studies have reported the application of Mn in sheep, poultry, and swine production. Wong-Valle et al. (1989) reported that Mn concentration of the liver, the kidney, and the bones of sheep based on the multiple linear regression slopes, and the relative bioavailability of Mn from MnO, MnO_2_, and MnCO_3_ averaged 57.7%, 32.9%, and 27.8%, compared with 100% for MnSO_4_. Manganese methionine hydroxyl analog chelated (Mn-MHAC; 25, 50, 75, and 100 mg Mn/kg) had a positive effect on body weight and average daily gain compared with the control group (Meng et al., 2021). Mn requirements for reproduction in swine are substantially greater than requirements for growth, with a recommendation of 25 mg/kg for gestating (Hurley and Keen, 1987). However, there are few reports about the application of manganese in calf production. Weaning impacts the trace mineral status of calves and likely influences health and productivity outcomes (Caramalac et al., 2017). Thus, adding trace elements to calf diets before and after weaning is a strategy to guarantee animal performance. Bampidis et al. (2021) reported that supplementing with Mn in the form of chelates of lysine and glutamic acid in a mixture of 1:1 (LGM) was an effective manganese source for animals and friendly to the environment. It is worth investigating in depth how the effect of LGM on microbiota and mineral elements of calves' feces pre-weaning and post-weaning, as compared with sulfate forms. It was hypothesized that supplementation with organic sources of Mn would improve nutrient digestion, increase the relative abundance of beneficial bacteria, and reduce the excretion of mineral elements to the environment during the pre-weaning and post-weaning. Thus, the present study assessed the influence of feeding organic or inorganic manganese on the apparent digestibility of nutrients, fecal bacteria, and mineral elements in feces in pre-weaning and post-weaning calves.

## 2. Materials and methods

### 2.1. Animals and experimental design

All animals were cared for according to protocols approved by the Yangzhou University Laboratory Animal Care and Use Committee.

**Figure 1:**
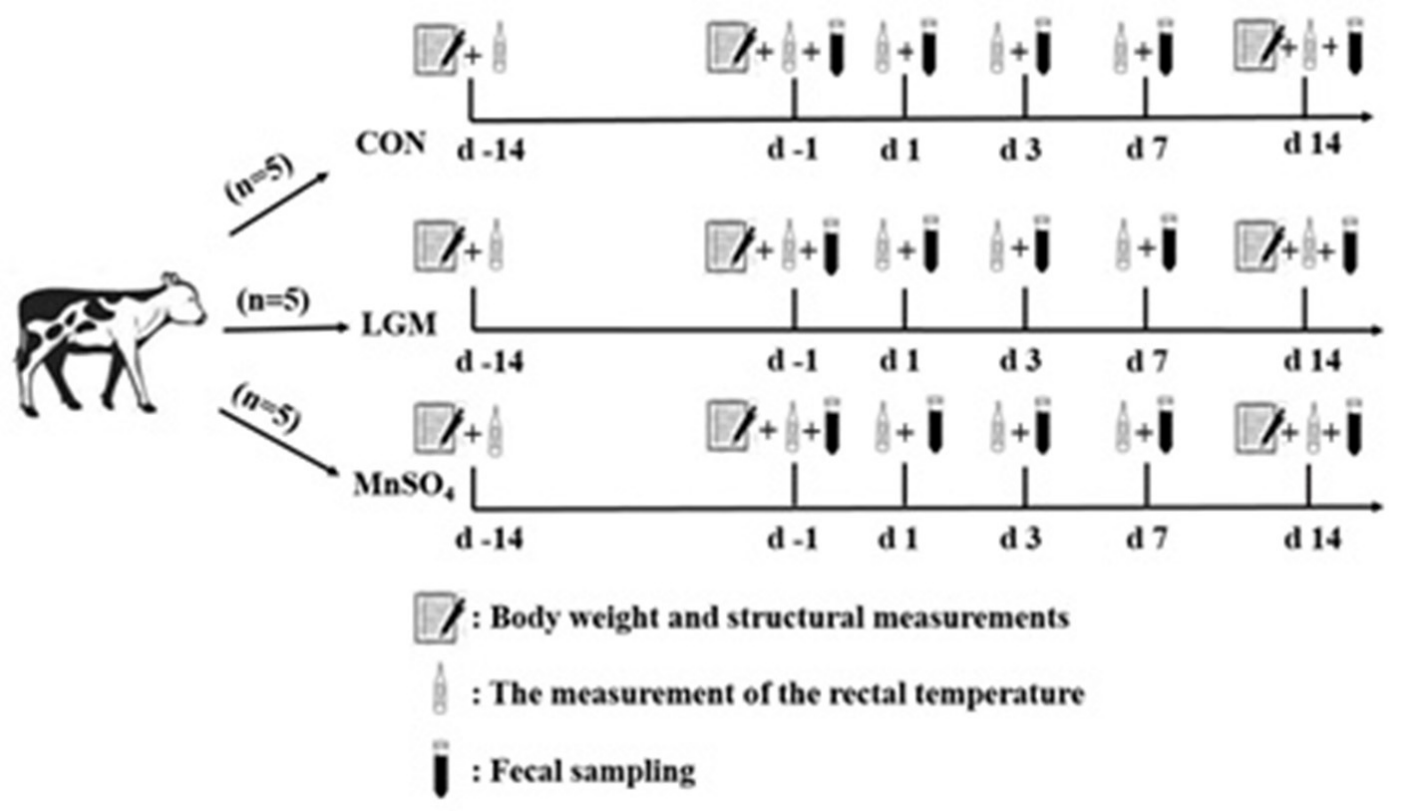
Diagram of the experimental design. LGM, in the form of chelates (lysine Mn: glutamic acid Mn = 1:1). MnSO_4_, in the form of sulfate Mn. days −14, −1, 1, 3, 7, and 14 represent calves at −14, −1, 1, 3, 7, and 14 days after weaning, respectively.

A total of 15 healthy Holstein calves (6 weeks old, 82.71 ± 1.35 kg BW) were randomly assigned to three groups and five calves each. Calves were housed individually and had free access to concentrate and water throughout the study period. The experimental diets were as follows: (1) a basal diet without Mn supplementation (CON), (2) 20 mg Mn/kg (dry matter basis) in the form of chelates (lysine Mn: glutamic acid Mn = 1:1, LGM), (3) 20 mg Mn/kg (dry matter basis) in the form of Mn sulfate (MnSO_4_; Figure 1). LGM was purchased from Zinpro Co. LTD (United States), and Mn content is 15%. Mn sulfate monohydrate (MnSO_4_·H_2_O) was purchased from Sichuan Combell Biotechnology Co. LTD (China), and Mn content is 31.8%. The study was conducted over a period of 28 days (28 November 2021 to 3 January 2022). Milk was provided (2 kg/ day), and concentrate plus oat hay was offered freely two times daily (08:00 h and 18:00 h). LGM and MnSO_4_ were mixed in milk pre-weaning and concentrate post-weaning. The chemical composition of the concentrate, oat hay, and milk fed to calves is shown in Supplementary Table 1.

### 2.2. Sampling and measurements

#### 2.2.1. Growth measurements

The amount of concentrate and oat hay offered and the residues were weighed daily for the average dry matter intake (DMI) calculation [average DMI/kg, (feed offered – residual feed)/ test days × DM%] of each calf. The calves were weighed at the beginning of the experiment (day −14), the last day before weaning (day −1), and the final day of the experiment (day 14) by electronic walk-over weighing scale at 2–3 h after the morning feeding. The body height (vertical distance between the highest point of the withers and the ground), body length (distance between the points of the shoulder and pin bone), chest girth (body circumference immediately behind the front shoulder at the fourth rib, posterior to the front legs), cannon circumference (horizontal circumference of the thinnest point on the cannon bone of the left forelimb), hip height (vertical distance between the highest point of the hook bone and the ground), and hip width (distance between the points of hook bones) were measured using a tape. The average daily gain [ADG/kg, (final body weight – initial body weight)/28], feed/gain (F/G/%, average DMI/ADG), and body size growth (final body size – initial body size) of calves overall (day −14 to day 14), pre-weaning (day −14 to day −1), and post-weaning [first day before weaning (day −1) to day 14] were calculated from the above data. The rectal temperature of a calf was measured on days −14, −1, 1, 3 after weaning (day 3), 7th day after weaning (day 7), and day 14. The results are shown in Supplementary Tables 2–4.

#### 2.2.2. Collection and measurements of fecal samples

Fecal samples were collected aseptically from calves at days −1, 1, 3, 7, and 14. In total, 10% diluted sulfuric acid was mixed with a fecal sample (2 ml per 100 g sample) for nitrogen fixation. All feed and fecal samples were placed in a 65°C oven until dried and grounded through a 0.35 mm screen for later determination. The fecal dry matter (DM), ash, organic matter (OM), ether extract (EE), and crude protein (CP) were determined following the method described by Silva and Queiroz (2006). Neutral detergent fiber (NDF) and acid detergent fiber (ADF) were analyzed following the method described by Soest (1994). In addition, acid insoluble ash (AIA) was used as an internal marker to determine the nutrient digestibility of nutrients which was calculated using the equation as follows:


$$\begin{array}{l} Nutrient\;digestibility\;\left(\%\right)\;=\;\left[1\;-\;\left(A1\;\times \;F2\right)\;/\;\left(A2\;\times \;F1\right)\right]\\ \times \;100\;according\;to \end{array}$$

Álvarez-Rodríguezetal et al. (2017).

where A1 and A2 are the nutrient contents in feces and diet (%), respectively, F1 and F2 are the AIA contents in feces and diet (%), respectively.

For analyzing bacterial community, the fresh fecal sample was collected and measured by high-throughput sequencing by Beijing Novogene Biotechnology Co., LTD. In brief, total bacterial DNA was extracted using a TIANαmp stool DNA kit (Tiangen Biotech Co., LTD). Then, the 16S rRNA V4 region gene was amplified by PCR using the primers 515F (5′-GTGCCAGCMGCCGCGGTAA-3′) and 806R (5′-GGACTACHVGGGTWTCTAAT-3′). Alpha-diversity (observed OTUs and α diversity index) and principal coordinate analysis (PCoA, unweighted Unifrac) were calculated using QIIME2.

For analyzing fecal minerals concentration, fresh samples at days −1, 1, 7, and 14 were placed in an oven at 45°C until dried. Then, 0.5 g samples were weighed into a 50 ml conical flask, digested (270°C) in the mixture of nitric acid and perchloric acid (6:1), and then diluted with deionized water to make a 25 ml solution. The minerals (iron, copper, calcium, phosphorus, manganese, and magnesium) were assayed by inductively coupled plasma emission spectroscopy (Optima 7300 DV, PerkinElmer Co., United States) (Esaka, 2017).

### 2.3. Statistical analysis

Data of apparent nutrient digestibility, fecal bacterial diversity indices, relative abundance of bacterial phylum and genus, and fecal mineral element concentrations were analyzed by PROC MIXED of SAS (version 9.4). The model used is as follows:


$$\begin{array}{l} {\text{Y}}_{\text{ijkl}}=\;\mu +\;{\text{T}}_{\text{i}}+\;{\text{R}}_{\text{j}}+\;{\text{e}}_{\text{ij}}+\;{\text{e}}_{\text{ijkl}} \end{array}$$

where Y_ijkl_ = observation, μ = general mean, T_i_ = treatment effect, R_j_ = block effect, e_ij_ = experimental error (block × treatment), and e_ijkl_ = sampling error (cow × block × treatment × sample).

The statistical significance of the means of apparent nutrient digestibility, Chao1, Shannon and Simpson indexes, the analysis of PCoA results, the relative abundance of bacteria, and the concentration of fecal minerals were defined by *P* < 0.05. It is worth noting that correlation coefficients with absolute values >0.5 are considered relevant. Significant differences were declared at *P* < 0.05, and *P* < 0.01 was considered a highly significant difference.

## 3. Results

### 3.1. Effects of different manganese sources on apparent nutrient digestibility of weaning calves

Table 1 shows the apparent nutrient digestibility for days −1, 1, 3, 7, and 14. Mn supplementation and the interaction of day × treatment significantly affected the apparent digestibility of nutrients (*P* < 0.01). Except for CP, there was a significant effect of days on the apparent nutrient digestibility (*P* < 0.05). The nutrient digestibility was the highest on day −1 in the LGM group. The nutrient digestibility was the highest on day 1 in the CON group (*P* < 0.01), and then gradually decreased with time. Apparent nutrient digestibility was greater in the LGM and MnSO_4_ groups than in the CON group on days 3 and 7 (*P* < 0.05). The apparent digestibility of DM, OM, CP, NDF, and ADF in the CON group was 45.26%−69.92%, 50.19%−73.08%, 42.38%−67.55%, 33.22%−55.80%, and 22.26%−34.90%, respectively. The values in the LGM group were 54.91%−64.89%, 60.14%−69.34%, 53.64%−63.05%, 44.38%−52.54%, and 25.19%−36.89%, and in the MnSO4 group, 51.17%−68.05%, 52.40%−72.54%, 40.05%−54.62%, 34.43–42.39%, and 23.74%−31.09%, respectively.

**Table 1:** Effects of different manganese sources on apparent digestibility of weaning calves.

**Item**	**Group**	**Duration of treatment (days)**					**SEM**	***P*** **-value**		
		−**1**	**1**	**3**	**7**	**14**		***D***	***T***	***D*** × ***T***
DM	CON	57.31^Cb^	69.92^Aa^	45.26^Ce^	47.13^Bd^	54.13^Bc^	7.19	< 0.01	< 0.01	< 0.01
	LGM	64.89^Ba^	64.60^Aa^	56.61^Bb^	57.61^Ab^	54.91^Ab^				
	MnSO_4_	68.05^Aa^	53.77^Bcd^	57.32^Abc^	57.95^Ab^	51.17^Bd^				
OM	CON	63.89^Bb^	73.08^Aa^	51.42^Bc^	50.19^Bc^	59.32^b^	6.92	< 0.01	< 0.01	< 0.01
	LGM	69.34^Aa^	67.25^Bb^	62.48^Ac^	62.75^Ac^	60.14^d^				
	MnSO_4_	72.54^Aa^	52.40^Cd^	62.50^Ab^	63.58^Ab^	56.89^c^				
CP	CON	49.57^bc^	67.55^Aa^	51.78^Bbc^	42.38^c^	54.31^Ab^	8.29	0.18	< 0.01	< 0.01
	LGM	63.05^a^	53.65^Bb^	53.64^Ab^	56.51^b^	56.26^Ab^				
	MnSO_4_	54.62	40.05^C^	52.50^A^	50.87	45.92^B^				
NDF	CON	43.74^Bb^	55.80^Aa^	33.22^Cc^	33.25^Cc^	38.69^bc^	6.72	< 0.01	< 0.01	< 0.01
	LGM	52.54^Aa^	44.95^Bb^	45.10^Ab^	44.38^Ab^	44.64^b^				
	MnSO_4_	42.39^B^	42.08^B^	39.34^B^	40.50^B^	34.43				
ADF	CON	25.35^Bb^	34.90^a^	24.20^b^	22.26^Bb^	22.76^b^	5.71	< 0.01	< 0.01	0.01
	LGM	36.89^Aa^	32.00^ab^	34.86^a^	26.47^Ab^	25.19^b^				
	MnSO_4_	28.77^B^	25.47	31.09	30.41^A^	23.74				

Values in the same row (a–e) or in the same column (A–C) with different letters are significantly different (P < 0.05). LGM, in the form of chelates (lysine Mn: glutamic acid Mn = 1:1). MnSO_4_, in the form of sulfate Mn. SEM, standard error of means. D, effect of day. T, effect of group. D × T, interaction between day and group.

### 3.2. Effects of different manganese sources on fecal microbiota diversity of weaning calves

In this study, 16S rRNA was amplified, and its sequence was analyzed to study the effects of supplementation on the fecal microbiota of calves pre-weaning and post-weaning at five time points (days −1, 1, 3, 7, and 14), with an average of 61,544 sequences per fecal sample. These results reflect the interaction between treatment and day in three alpha diversity indices (Table 2, *P* < 0.05). The Chao 1 index of CON is significantly higher than that of LGM and MnSO_4_ on day −1 (*P* < 0.05), and then gradually decreases. However, in the LGM and MnSO_4_ groups, it was stabilized from days −1 to 14. The Shannon and Simpson index was significantly higher in the CON and LGM groups than the MnSO_4_ group on day 1 (*P* < 0.05).

**Table 2:** Effects of different manganese sources on fecal bacterial Chao1, Shannon, and Simpson indexes of weaning calves.

**Item**	**Group**	**Duration of treatment (days)**					**SEM**	***P*** **-value**		
		−**1**	**1**	**3**	**7**	**14**		***D***	***T***	***D*** × ***T***
Chao1	CON	887.36^A^	889.12	759.12	730.27	617.50	123.53	0.28	0.04	0.04
	LGM	696.57^B^	681.35	737.62	717.54	707.20				
	MnSO_4_	704.88^B^	653.97	714.67	735.71	699.50				
Shannon	CON	7.80	7.71^A^	7.33	7.37	7.01	0.65	0.47	0.01	0.03
	LGM	7.55	7.54^A^	7.49	7.12	7.44				
	MnSO_4_	7.20	6.19^B^	6.91	7.49	7.15				
Simpson	CON	0.99	0.99^A^	0.98	0.98	0.98	0.04	0.21	< 0.01	0.01
	LGM	0.99	0.99^A^	0.98	0.97	0.98				
	MnSO_4_	0.95	0.88^B^	0.95	0.98	0.98				

Values in the same column (A, B) with different letters are significantly different (P < 0.05). LGM, in the form of chelates (lysine Mn: glutamic acid Mn = 1:1). MnSO_4_, in the form of sulfate Mn. SEM, standard error of means. D, effect of day. T, effect of group. D × T, interaction between day and group.

**Figure 2:**
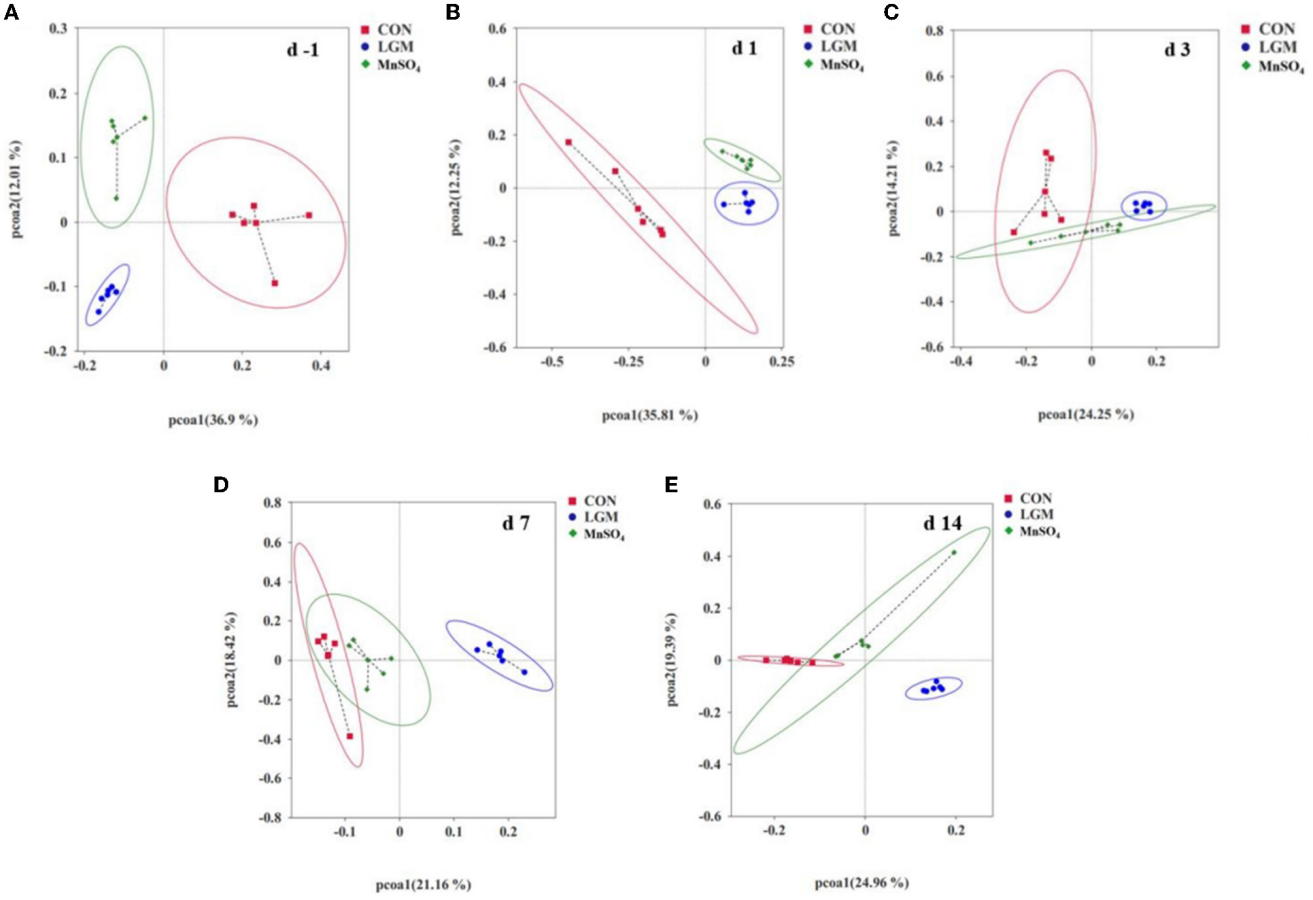
Based on UniFrac principal coordinates analysis plots (PCoA). Panels **(A–E)** indicate days −1, 1, 3, 7, and 14 bacteria PCoA pictures. Data are shown as the percentages of the total identified sequences per group. days −1, 1, 3, 7, and 14 represent calves at −1, 1, 3, 7, and 14 days after weaning, respectively. PCoA 1 and PCoA represent principal components 1 and 2, respectively. LGM, in the form of chelates (lysine Mn: glutamic acid Mn = 1:1). MnSO_4_, in the form of sulfate Mn.

The principal coordinates analysis plot results showed that the fecal microbial community on day −1 was significantly different among the three groups (Figure 2A). On day 1, the community of LGM and MnSO_4_ was close (Figure 2B). By contrast, the community of MnSO_4_ closed to CON on days 3, 7, and 14 after weaning, and that of LGM was kept more distance from the CON group (Figures 2C–E).

### 3.3. Effects of different manganese sources on the relative abundance of fecal microbiota in weaning calves

**Figure 3:**
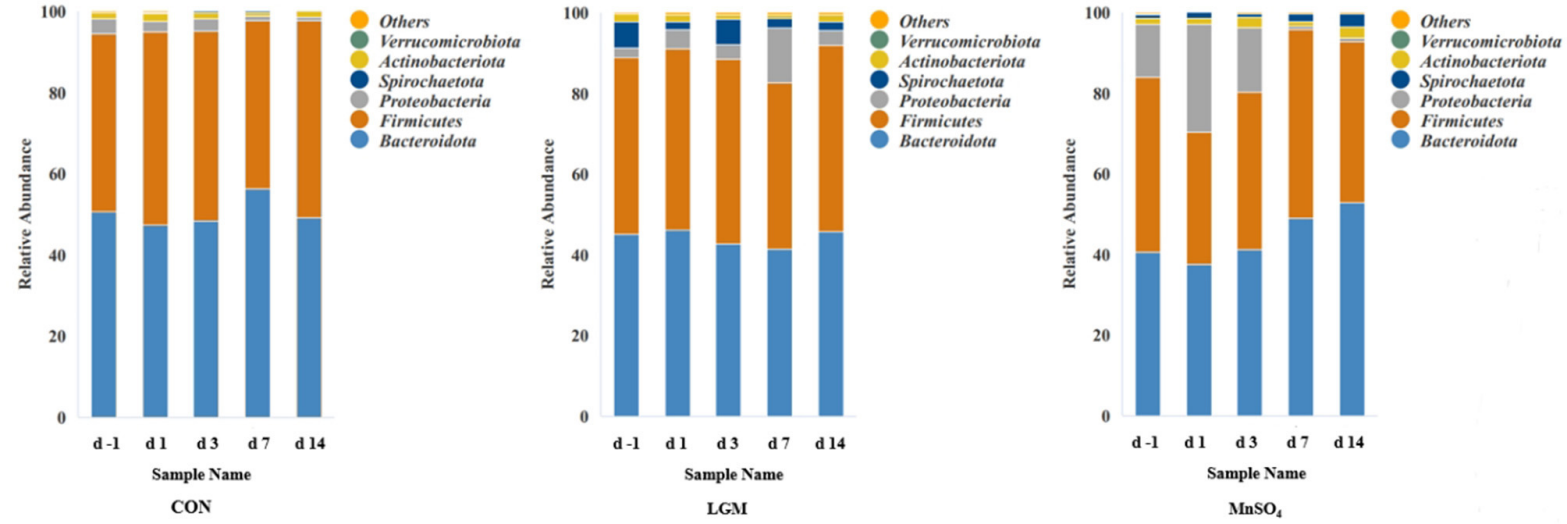
Relative abundances of the major phylum fecal bacterial. Data are shown as the percentages of the total identified sequences per group. days −1, 1, 3, 7, and 14 represent calves at −1, 1, 3, 7, and 14 days after weaning, respectively. LGM, in the form of chelates (lysine Mn: glutamic acid Mn = 1:1). MnSO_4_, in the form of sulfate Mn.

The top six relative abundances of fecal bacteria at the phylum level were analyzed (Figure 3 and Supplementary Table 5). The predominant bacteria were *Bacteroidota* and *Firmicutes*. There was no significant difference in the relative abundance of *Bacteroidota* and *Actinobacteriota* according to Mn source or days. No day effect was observed in the relative abundance of *Firmicutes* and *Verrucomicrobiota*. Compared with CON, the relative abundance of *Firmicutes* in MnSO_4_ was lower on days −1, 7, and 14 (*P* < 0.01), and that in LGM was lower on day 7 (*P* < 0.01). The relative abundance of *Proteobacteria* was higher on day −1 in the MnSO_4_ group and day 14 in the LGM group (*P* < 0.01). It decreased in the MnSO_4_ group on days 7 and 14 compared with days −1, 1, and 3 (*P* = 0.02). The relative abundance of Spirochaetota in the LGM group was significantly higher than that of the CON and MnSO_4_ groups at days −1 and 3 (*P* < 0.05).

The interaction of day × treatment was significant (*P* < 0.05) for the relative abundance of *Alloprevotella, Prevotellaceae*_UCG-001, *Clostridia* UCG 014, RF39, UCG-010, *Pseudomonas, Ralstonia*, and *Treponema* (*P* < 0.05; Table 3). Day effect was observed in the relative abundance of seven genera (*Prevotella, Alloprevotella, Alistipes*, UCG-005, *Clostridia* UCG 014, *Pseudomonas*, and *Ralstonia*; *P* < 0.05). The treatment effect was observed in the relative abundance of all genera (*P* < 0.05). The genus *Bacteroides*, which belongs to Bacteroidota, was the most abundant in the three groups.

**Table 3:** Effects of different manganese sources on fecal bacteria of weaning calves at the genus level.

**Phylum**	**Genus**	**Group**	**Duration of treatment (days)**					**SEM**	***P*** **-value**		
			−**1**	**1**	**3**	**7**	**14**		***D***	***T***	***D*** × ***T***
*Bacteroidota*	*Bacteroides*	CON	18.59^A^	23.41^A^	23.07	16.44^A^	17.68	6.89	0.80	< 0.01	0.15
		LGM	11.65^B^	12.39^B^	11.37	10.42^B^	12.38				
		MnSO_4_	10.99^Bbc^	9.57^Bc^	12.58^abc^	16.47^Aab^	18.58^a^				
	*Prevotella*	CON	1.40^Bb^	2.22^Bb^	3.11^b^	3.13^b^	5.92^Ba^	2.28	< 0.01	< 0.01	0.77
		LGM	1.98^Bb^	1.49^Bb^	2.68^b^	2.50^b^	4.73^Ba^				
		MnSO_4_	4.13^A^	3.31^A^	4.79	6.60	7.59^A^				
	*Alloprevotella*	CON	2.24^Ab^	2.56^Ab^	2.19^Ab^	2.78^Ab^	5.03^Aa^	1.32	< 0.01	< 0.01	< 0.01
		LGM	0.40^B^	0.39^B^	0.39^B^	0.55^B^	0.81^C^				
		MnSO_4_	0.57^Bb^	0.32^Bb^	1.60^ABa^	2.30^Aa^	2.48^Ba^				
	*Alistipes*	CON	4.80^Aa^	2.97^Ab^	2.68^b^	2.13^b^	1.84^b^	1.27	< 0.01	< 0.01	0.22
		LGM	4.33^A^	3.83^A^	2.75	3.10	2.58				
		MnSO_4_	2.91^B^	1.50^B^	1.81	1.76	2.18				
	*Bacteroidales RF16 group*	CON	0.26^B^	0.27^B^	0.15	0.35	0.15^B^	1.41	0.90	< 0.01	0.53
		LGM	1.74^A^	1.37^A^	1.68	1.58	2.60^A^				
		MnSO_4_	1.56^A^	2.75^A^	1.35	2.26	1.28^AB^				
	*Rikenellaceae RC9 gut group*	CON	1.00^B^	1.02^C^	1.00^B^	1.31^B^	1.99	1.92	0.22	< 0.01	0.25
		LGM	3.48^A^	4.05^A^	4.61^A^	5.05^A^	3.76				
		MnSO_4_	2.77^AB^	2.42^B^	2.86^AB^	2.38^B^	4.98				
	*Prevotellaceae_UCG-001*	CON	0.28^B^	0.53	0.43^B^	0.59	0.45	0.56	0.17	< 0.01	0.04
		LGM	1.93^A^	0.88	0.78^A^	0.89	0.71				
		MnSO_4_	0.67^B^	0.65	0.57^AB^	0.58	0.53				
*Firmicutes*	*UCG-005*	CON	8.53^A^	9.09^A^	8.35	12.93	7.55	3.61	0.05	< 0.01	0.17
		LGM	7.18^A^	6.30^B^	7.40	5.65	4.87				
		MnSO_4_	4.35^Bb^	3.41^Cb^	3.83^b^	8.63^a^	5.50^b^				
	*Clostridia UCG 014*	CON	3.05^Bc^	3.34^Bc^	4.20^Bbc^	4.80^b^	6.40^a^	1.60	< 0.01	< 0.01	0.02
		LGM	4.36^Bb^	5.33^Aab^	3.92^Bb^	4.50^ab^	6.09^a^				
		MnSO_4_	6.67^A^	4.86^A^	6.44^A^	6.04	7.37				
	*RF39*	CON	2.81	2.80^AB^	2.84	2.57	2.81	1.14	0.58	0.79	0.01
		LGM	3.60^ab^	4.17^Aa^	1.96^c^	2.32^bc^	2.45^bc^				
		MnSO_4_	2.46^ab^	1.62^Bb^	2.99^ab^	2.61^ab^	3.82^a^				
	*UCG-010*	CON	1.22	1.44^A^	1.37^A^	2.68^A^	2.30^A^	0.88	0.07	< 0.01	0.05
		LGM	1.18	1.64^A^	1.17^A^	1.41^AB^	0.56^B^				
		MnSO_4_	0.57	0.37^B^	0.42^B^	0.90^B^	1.00^B^				
	*Sharpea*	CON	0.03	0.05	0.12	0.04^B^	0.08	0.52	0.21	0.01	0.62
		LGM	0.07	0.08	0.06	0.14^B^	0.27				
		MnSO_4_	0.29	0.07	0.40	0.49^A^	1.07				
*Proteobacteria*	*Pseudomonas*	CON	0.13^C^	0.24^C^	0.11	0.16^B^	0.30^B^	1.85	0.03	< 0.01	< 0.01
		LGM	1.63^B^	2.36^B^	2.38	2.65^A^	2.59^A^				
		MnSO_4_	4.15^Aab^	4.78^Aa^	2.95^b^	0.05^Bc^	0.14^Bc^				
	*Ralstonia*	CON	0.28	0.03^B^	0.20	0.02^B^	0.01^B^	0.66	< 0.01	< 0.01	< 0.01
		LGM	0.31	0.88^AB^	0.52	0.63^A^	0.46^A^				
		MnSO_4_	0.88^b^	1.85^Aa^	0.62b	0.06^Bb^	0.03^Bb^				
*Spirochaetota*	*Treponema*	CON	0.02^B^	0.09^A^	0.07^B^	0.07	0.06	2.50	0.11	< 0.01	< 0.01
		LGM	6.34^Aa^	1.94^Ab^	6.39^Aa^	2.36^b^	2.17^b^				
		MnSO_4_	1.04^B^	1.32^B^	0.90^B^	2.08	3.15				
*Actinobacteriota*	*Bifidobacterium*	CON	0.74	0.24	0.72^B^	0.16	0.24	1.43	0.49	0.07	0.91
		LGM	0.19	0.07	0.21^B^	0.18	0.95				
		MnSO_4_	0.77	0.96	1.76^A^	0.44	2.13				
	*Olsenella*	CON	0.31^B^	0.89	0.53	0.62	0.79	0.66	0.22	< 0.01	0.15
		LGM	1.53^A^	1.48	0.52	0.54	0.63				
		MnSO_4_	0.49^B^	0.27	0.35	0.21	0.42				
*Verrucomicrobiota*	*Akkermansia*	CON	0.01^B^	0.01	0.00	0.00^B^	0.00	0.07	0.20	< 0.01	0.65
		LGM	0.11^A^	0.13	0.05	0.02^A^	0.06				
		MnSO_4_	0.04^B^	0.01	0.00	0.00^B^	0.00				

Values in the same row (a–c) or in the same column (A–C) with different letters are significantly different (P < 0.05). LGM, in the form of chelates (lysine Mn: glutamic acid Mn = 1:1). MnSO_4_, in the form of sulfate Mn. SEM, standard error of means. D, effect of day. T, effect of group. D × T, interaction between day and group.

### 3.4. Effects of different manganese sources on fecal minerals excretion of weaning calves

There was a significant difference in the concentration of minerals according to days (Table 4, *P* < 0.01). No treatment effect was observed in the Fe concentration. Moreover, the interaction of day × treatment was shown in the concentrations of Cu, P, Mn, and Mg (*P* < 0.01). The decrease in Fe, Ca, and P concentrations with days was observed in the two groups (*P* < 0.01). The concentration of Cu and Mg was higher on day 1 in the LGM group and day −1 in the MnSO_4_ group (*P* < 0.01). The Mn concentration was significantly lower on day −1 in the LGM and MnSO_4_ groups than at other time points (*P* = 0.01).

**Table 4:** Effects of different manganese sources on fecal minerals excretion of weaning calves.

**Item**	**Group**	**Duration of treatment (days)**				**SEM**	***P*** **-value**		
		**–1**	**1**	**7**	**14**		***D***	***T***	***D*** × ***T***
Fe	LGM	801.47^a^	504.15^b^	586.69^b^	380.27^c^	178.16	< 0.01	0.12	0.12
(mg/kg)	MnSO_4_	849.46^a^	591.63^b^	508.63^b^	552.70^b^				
Cu	LGM	75.36^b^	82.41^a^	75.96^b^	61.39^c^	8.26	< 0.01	< 0.01	< 0.01
(mg/kg)	MnSO_4_	73.90^a^	66.17^b^	67.46^b^	59.09^c^				
Ca	LGM	1.92	1.65	1.59	1.45	0.10	0.02	< 0.01	0.22
(%)	MnSO_4_	1.92^a^	1.71^a^	0.68^b^	0.76^b^				
P	LGM	0.80	0.73	0.71	0.74	0.47	< 0.01	< 0.01	< 0.01
(%)	MnSO_4_	1.02^a^	0.87^b^	0.81^b^	0.77^b^				
Mn	LGM	435.24^c^	591.94^a^	586.99^a^	484.01^b^	75.33	< 0.01	0.01	< 0.01
(mg/kg)	MnSO_4_	469.44^c^	540.95^b^	663.31^a^	526.16^b^				
Mg	LGM	4651.46^b^	5218.06^a^	4658.62^b^	4196.49^c^	368.52	< 0.01	< 0.01	< 0.01
(mg/kg)	MnSO_4_	5322.88^a^	5115.52^b^	4998.34^bc^	4848.35^c^				

Values in the same row (a–c) with different letters are significantly different (P < 0.05). LGM, in the form of chelates (lysine Mn: glutamic acid Mn = 1:1). MnSO_4_, in the form of sulfate Mn. SEM, standard error of means. D, effect of day. T, effect of group. D × T, interaction between day and group.

### 3.5. Correlation analysis of fecal minerals and fecal major bacteria

**Figure 4:**
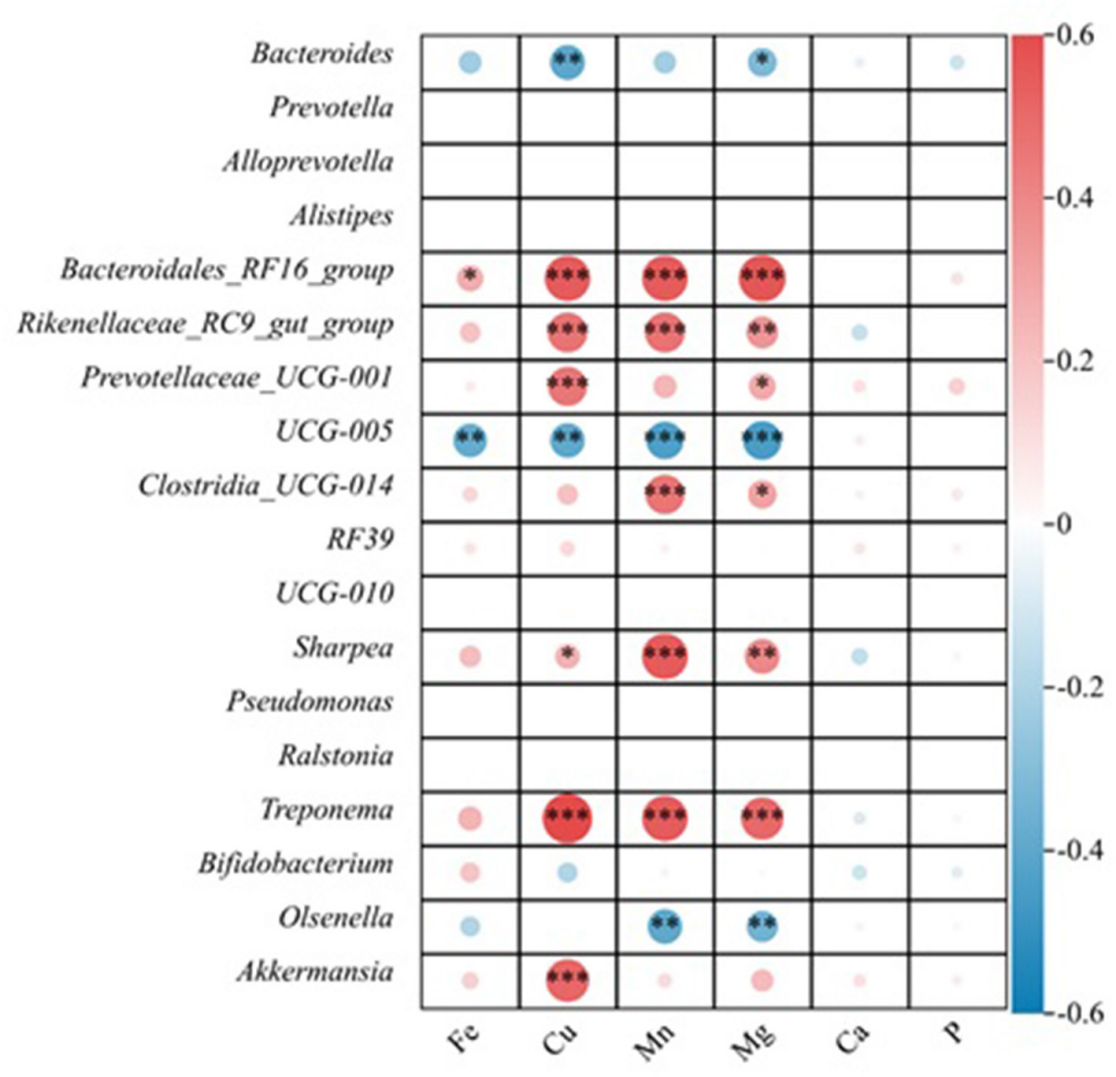
Spearman's correlation between fecal minerals and bacterial population. The area of the circle indicates the magnitude of the correlation, and the different colors indicate a positive correlation (red) or negative correlation (blue). * indicates 0.01 < *P* ≤ 0.05, ** indicates *P* ≤ 0.01, and *** indicates *P* ≤ 0.001.

Spearman's correlation analysis was performed to assess the relationship between fecal minerals and fecal microbiota structure in the presence of manganese sources. At the genus level, the relative abundance of *Bacteroides* was negatively correlated with the concentration of Cu (*r* = −0.40, *P* < 0.01) and Mg (*r* = −0.32, *P* < 0.05). The relative abundance of *UCG-005* was negatively correlated with the concentrations of Fe (*r* = −0.38, *P* < 0.01), Cu (*r* = −0.40, *P* < 0.01), Mn (*r* = −0.44, *P* < 0.01), and Mg (*r* = −0.45, *P* < 0.01). The relative abundance of *Olsenella* was negatively correlated with the concentration of Mn (*r* = −0.40, *P* < 0.01) and Mg (*r* = −0.35, *P* < 0.01). The concentration of Fe was positively correlated with the relative abundance of the *Bacteroidales_RF16_group* (*r* = 0.28, *P* < 0.05). The concentration of Cu was positively correlated with the relative abundance of *Bacteroidales_RF16_group* (*r* = 0.55, *P* < 0.001), *Rikenellaceae_RC9_gut_group* (*r* = 0.47, *P* < 0.001), *Prevotellaceae_UCG-001* (*r* = 0.46, *P* < 0.001), *Sharpea* (*r* = 0.26, *P* < 0.05), *Treponema* (*r* = 0.61, *P* < 0.001), and *Akkermansi*a (*r* =0.51, *P* < 0.001). The concentration of Mn was positively correlated with the relative abundance of *Bacteroidales_RF16_group* (*r* = 0.55, *P* < 0.001), *Rikenellaceae_RC9_gut_group* (*r* = 0.47, *P* < 0.001), *Clostridia_UCG-014* (*r* = 0.46, *P* < 0.001), *Sharpea* (*r* = 0.56, *P* < 0.001), and *Treponema* (*r* = 0.55, *P* < 0.001). The concentration of Mg was positively correlated with the relative abundance of *Bacteroidales_RF16_group* (*r* = 0.57, *P* < 0.001), *Rikenellaceae_RC9_gut_group* (*r* = 0.36, *P* < 0.01), *Prevotellaceae_UCG-001* (*r* = 0.29, *P* < 0.05), *Clostridia_UCG-014* (*r* = 0.31, *P* < 0.05), *Sharpea* (*r* = 0.40, *P* < 0.01), and *Treponema* (*r* = 0.51, *P* < 0.001; Figure 4).

## 4. Discussion

This study was based on the laboratory's previous dietary addition of 40 mg Mn/kg to Holstein cows (milk yield: 39.64 ± 5.55 kg, body weight: ~668.0 kg), which promoted both feed intake and milk production of dairy cows. In addition, the manganese absorption rate of calves is higher than that of adult animals. Therefore, 20 mg Mn/kg was selected to be added into calf diets to study the effects of this concentration on the apparent digestibility of nutrients, fecal microorganisms, and excretion of fecal mineral elements of calves.

### 4.1. Effects of different manganese sources on apparent digestibility of weaning calves

The digestibility of nutrients reflects the utilization of nutrients by animals, which is important for animal growth. Insufficient solid feed intake results in reduced apparent digestibility of nutrients, especially NDF and ADF, thus it is assumed that low solid feed intake may reduce the growth rate of calves post-weaning (Hill et al., 2010). In this study, the DMI of overall and post-weaning was significantly higher in the LGM and MnSO_4_ groups than in the CON group. Due to the low feed intake in the CON group, the apparent digestibility of DM in this group was lower than that of the LGM group except for day 1, indicating that the addition of LGM improved the nutrient utilization of calves. The diets supplemented with Mn, Zn, and Cu as methionine, glycine, and sulfate salts, respectively, had positive effects on DMI in dry cows (Roshanzamir et al., 2020). In the other study, sulfate sources reduced the total digestibility of NDF in cows compared with hydroxy sources in Cu, Mn, and Zn (Faulkner and Weiss, 2017). The results of this study are consistent with the study by Faulkner and Weiss (2017), and this may indicate stronger digestion and absorption of calves in the organic mineral-supplemented group. In addition, we observed a decrease in digestibility in calves post-weaning. Weaning stress was ruled out as it was not observed throughout the trial period. We speculated that the calves have an increased feed intake after weaning, and only solid feed was available.

### 4.2. Effects of different manganese sources on the microbiota diversity and relative abundance of feces of weaning calves

The unique OTU number of the CON group in this study was higher than that of the LGM and MnSO_4_ groups at −1, 1, 3, and 7 days of weaning and decreased on day 14. The specific OTU number of the LGM and MnSO_4_ groups had been steadily increasing and exceeded the CON group on day 14. The analysis within-group revealed that the specific OTU number of the LGM and MnSO_4_ groups was proportional to the increase in age, but this was not found in the CON group. The Chao 1 index was numerically increased in the LGM and MnSO_4_ groups on days 3 and 7, which hinted that the bacterial richness of calves was disordered by weaning, but it could be quickly repaired by supplementation with Mn. The Shannon index was also not affected by time factors, but a lower Shannon index was observed in the LGM and MnSO_4_ groups. The Shannon index represented bacterial diversity and evenness (Shabat et al., 2016). Combining the digestibility and growth performance of calves, the lower the microbiota diversity in the rumen is accomplished by the higher metabolic efficiency (Tuomisto, 2010). However, calves fed with extra manganese may provide more digestible fermentation products for animals in this study. According to previous reports, the stable equilibrium of microbial community lies in the ability as a whole to restore such changes to the original status when individual bacteria are disturbed or temporally damaged (Lozupone et al., 2012). A characteristic of the establishment of a healthy human gut microbiota is the increase in early diversity and stability of the microbiota (Palmer et al., 2007; Yatsunenko et al., 2012). In this study, the bacterial community of LGM did not coincide with that of CON and over the entire study, which indicated that organic Mn resulted in a more aggregated and stable bacterial community. The addition of LGM to the calf diet may have the function of maintaining the fecal microbiota during the weaning period.

Calves were fed solid feed pre-weaning, and there was no effect of time on the bacterial community at the phylum level, suggesting that the microbial community developed more maturely with the aging of calves (Li et al., 2012). Meale et al. (2016) also found that different weaning strategies (gradual vs. abrupt) did not affect the fecal microbiota of pre-weaning and post-weaning calves if they were fed solid feed before weaning. Research showed that the developing gut (1-day-old−2-month-old) and the more mature gut (2-year-old) contain the same dominant bacteria (*Bacteroidota, Firmicutes*, and *Proteobacteria*), but the relative abundance changes depending on the stage of development (Jami et al., 2013). Jami et al. (2013) reported that Bacteroidota was the predominant phylum of bacteria in 2-month-old calves, and the results obtained in this study are consistent with the study by Jami. According to published reports, *Prevotellaceae*_UCG-001 has the ability to degrade non-cellulosic polysaccharides, pectins, and proteins (Flint et al., 2008; Kabel et al., 2011). The results of this study showed that the relative abundance of UCG-005 and UCG*-*010 was higher in the CON group. This may be the reason for the higher relative abundance of *Firmicutes* in the CON group than in the MnSO_4_ group on days 1, 7, and 14. It was found that Ruminococcaceae genera (UCG-005 and UCG-010) can convert complex polysaccharides into a variety of nutrients for the host (La Reau and Suen, 2018). *Sharpea* act as an important factor of lactate production and utilization (Kamke et al., 2016). In the present study, we observed an increase in the relative abundance of *Sharpea* in the MnSO_4_ group on day 7, although the relative abundance is not influenced by the time factor. Myer et al. (2015) detected that many genera in *Firmicutes* were associated with high ADG, consistent with the results of the present study.

The relative abundance of *Pseudomonas* and *Ralstonia* in the LGM group was higher than the other two groups. Proteobacteria is the dominant phylum in many environmental ecological niches (Lauber et al., 2009; Redford and Fierer, 2009) and play an important role in the colonization of the intestinal anaerobes of newborn calves. In this study, the relative abundance of *Proteobacteria* in the CON and MnSO_4_ groups was significantly higher than that of the LGM group on day −1 and then gradually decreased. However, in the LGM group, it was stabilized from days −1 to 14 and significantly higher on day 14 than the other two groups. In addition, the organic mineral-supplemented group had a significantly higher relative abundance of Spirochaetota than the CON and MnSO_4_ groups on days −1, 1, and 3. The richness of *Treponema*, a member of Spirochaetota, was significantly higher in the LGM group than that of the CON and MnSO_4_ groups at −1, 1, and 3 days too. Gharechahi and Salekdeh (2018) performed metagenomic sequencing of rumen microorganisms of grazing camels and showed that Spirochaetota has significant potential to contribute to cellulose and hemicellulose degrading enzymes. Members of Spirochaetota, especially *Treponema*, are associated with the degradation of pectin in the rumen (Liu et al., 2015). The role played by Verrucomicrobiota in the rumen has been underappreciated due to its uncultured *in vitro* nature. It was found that Verrucomicrobiota is an organism known to code a variety of carbohydrate-degrading enzymes, peptidases, and sulfatase and is therefore considered well suited for degrading lignocellulose in the rumen (Martinez-Garcia et al., 2012). As observed in this study, the organic mineral group showed a more relative abundance of Verrucomicrobiota on day −1. In addition, the relative abundance of *Akkermansia*, which belonged to the Verrucomicrobiota phylum, was significantly higher in the LGM group on days −1 and 7. These results indicated that calves in the organic manganese group may have a stronger ability to degrade lignocellulose than those in the CON and MnSO_4_ groups. In addition, this also echoes the fact that calves in the LGM group had higher apparent digestibility of NDF and ADF.

### 4.3. Effects of different manganese sources on fecal trace minerals of weaning calves

In nature, trace minerals generally exist in the ionic state or in combination with certain molecular ligands (Weller et al., 2014), and this property is essential for animals, making them highly necessary components in animal diets. They excrete the excess supply through the circulation of animal organisms, and feces is one of the routes of excretion (Windisch, 2002). The concentrations of Fe, Cu, Ca, P, Mn, and Mg in the feces of lactating cows were 879 mg/kg, 75.7 mg/kg, 2.65%, 0.76%, 311 mg/kg, and 0.99%, respectively (Sheppard and Sanipelli, 2012). The results of this experiment were slightly lower than those of the Sheppard study except for Mn, which may be due to the lower intake of calves compared with lactating cows. Moreover, the concentration of trace minerals in feces was three times in the feed, which is consistent with the results of the present study (Sheppard et al., 2010). The absorption of Mn in cows is low (0.75% reported by NRC), and therefore, Mn is excreted in excess through the bile and small intestine (Ho et al., 1984). In the current study, the content of Mn in feces increased with the intake of calves, and as the Mn content in feces increased, the Fe content gradually decreased. Fe is the most important cofactor for oxygen transport and transfer in living organisms. Non-heme iron in the diet mainly exists as Fe^3+^, which needs to be reduced to Fe^2+^ by ferroreductase, and then transported across the intestinal epithelium by divalent metal ion transporter 1 (DMT1) (Gómez et al., 2005). DMT1 can also transport other metal ions such as Cu and Mn through a proton coupling mechanism (Gómez et al., 2005). However, sharing a common pathway in intestinal absorption through DMT1 results in the concentration of Mn affecting Fe and Cu absorption and utilization (Arredondo et al., 2003; Garrick et al., 2003). Data from this experiment suggest that the amount of Cu in feces, which is similar to Fe, showed a pattern of increasing with the amount of fecal Mn in the mineral-supplemented groups. Ca is absorbed in the GIT by metabolism-driven intercellular transport and paracellular pathway (Liu et al., 2022). Fe can reduce the bioavailability of Ca (Zhang and Liu, 2022), and in this study, the fecal calcium and iron in the LGM and MnSO_4_ groups showed a consistent pattern, with the highest concentration on day −1 and the lowest concentration on day 14. Overall, fecal calcium concentrations were not affected by the interaction. The P contents in the feces of 4-month-old heifers were lower (0.54%) than that in the present study (Bjelland et al., 2011), probably due to different basal diets for calves. The net absorption of Mg in the small intestine and large intestine is relatively low (Care and Vantklooster, 1965), which may be the fact that Mg excretion in the feces is higher than the other trace elements.

### 4.4. Correlation analysis of fecal minerals and fecal major bacteria

Carrothers et al. (2015) analyzed the relationship between the fecal microbiota of lactating women and their diet and found that the intake of manganese was positively correlated with the relative abundance of *Firmicutes* (*r* = 0.44), and the relative abundance of *Bacteroides* was negatively correlated (*r* = −0.48). *Clostridia_UCG-014* and *Sharpea* belonged to *Firmicutes*, and their relative abundance was positively correlated with the concentration of Mn in this study. *Sharpea* is a lactic acid-producing bacterium, and it has been reported that high concentrations of Mn^2+^ are required for the growth of lactic acid bacteria (Archibald and Fridovich, 1981; Kamke et al., 2016). In addition, this study differs from that of Carrothers et al. (2015). First, *UCG-005* in *Firmicutes* was negatively correlated with the concentration of Mn, which may be because the relative abundance of this genus was higher in the group without an Mn source. Second, the relative abundance of *Bacteroidales_RF16_group* and *Rikenellaceae_RC9_gut_group* in the Bacteroides phylum was positively correlated with the concentration of Mn. This may be due to the fact that the experimental animals in this study are different from the study by Carrothers et al. (2015). The experimental animals in this study were 2-month-old calves, and the dominant bacterial genus of calves during this period was *Bacteroidetes* (Jami et al., 2013).

## 5. Conclusion

The addition of LGM pre-weaning and post-weaning increased the DMI, ADG, and chest circumference compared with the control group and the inorganic Mn group as the sulfate salt. The digestibility of pre-weaning and post-weaning calves in the LGM group was not greatly affected. In addition, LGM altered the fecal microbiota, and the relative abundance of the fiber-degrading bacteria *Treponema* and *Akkermansia* increased and a more stable fecal microbiota of pre-weaning and post-weaning calves in the LGM group. LGM increased the deposition of Mn in the body, which was beneficial to environmental protection in terms of fecal mineral element excretion. Based on the factors of fecal bacterial community and environmental protection, adding 20 mg/kg LGM to the diet containing 158.82 mg/kg Mn is the best.

## Data availability statement

The datasets presented in this study can be found in online repositories. The names of the repository/repositories and accession number(s) can be found below: https://ngdc.cncb.ac.cn/gsa/browse/CRA009807.

## Ethics statement

The animal study was reviewed and approved by Yangzhou University, the Institutional Animal Care and Use Committee.

## Author contributions

HJ and ML performed the experiment and included chemical analysis, statistical analysis, and manuscript writing. ML and JZ worked on the manuscript revision and gave valuable advice. DT, ZC, and YC conducted the study. All authors have read and approved the final version of this manuscript.
