# Supplementary material for: Effects of different manganese sources on nutrient digestibility, fecal bacterial community, and mineral excretion of weaning dairy calves
Source: Front Microbiol. 2023 May 18;14:1163468. doi: 10.3389/fmicb.2023.1163468 (PMC10232960; doi:10.3389/fmicb.2023.1163468)
Supplement: Supplementary file 1 [file Table_1.pdf]

Table 1 The chemical compositions and minerals content of the concentrate and oat hay, and minerals content of milk

Item	Concentrate	Oat hay	Milk
DM (%)	88.19	90.57	—
Ash (%)	7.60	7.19	—
CP (%)	19.83	6.63	—
EE (%)	23.27	14.31	—
NDF (%)	14.61	55.87	—
ADF (%)	6.4	33.21	—
Ca (%)	0.62	0.47	9.94
P (%)	0.62	0.28	11.48
Mg (mg/kg)	2040.14	1221.45	1.73
Fe (mg/kg)	305.84	92.90	0.02
Cu (mg/kg)	19.50	4.33	—
Mn (mg/kg)	158.82	98.47	—
