# Supplementary material for: Effects of different manganese sources on nutrient digestibility, fecal bacterial community, and mineral excretion of weaning dairy calves
Source: Front Microbiol. 2023 May 18;14:1163468. doi: 10.3389/fmicb.2023.1163468 (PMC10232960; doi:10.3389/fmicb.2023.1163468)
Supplement: Supplementary file 2 [file Table_2.pdf]

Table 2 Effects of different manganese sources on average daily gain, feed intake and feed/gain of calves

Item	CON	LGM	$MnSO_4$	SEM	*P*-value
Birth weight (kg)	40.10	40.38	41.92	0.51	0.31
Initial body weight (kg)	82.50	85.50	80.70	1.35	0.39
Final body weight (kg)	110.00	117.13	109.20	1.90	0.21
Overall ADG (kg)	0.99	1.09	1.07	0.03	0.26
Weaning weight (kg)	93.90	97.88	95.50	1.54	0.63
Pre - weaning ADG (kg)	0.82	0.83	0.98	0.05	0.37
Post - weaning ADG (kg)	1.17^{b}	1.38^{a}	1.17^{b}	0.04	0.01
Overall feed intake (kg)	2.36^{c}	3.06^{b}	3.21^{a}	0.03	<0.01
Pre - weaning feed intake (kg)	2.05^{c}	2.53^{b}	2.87^{a}	0.04	<0.01
Post - weaning feed intake (kg)	2.70^{b}	3.62^{a}	3.59^{a}	0.04	<0.01
Overall-F/G	2.42^{b}	2.80^{ab}	3.05^{a}	0.11	0.03
Pre - weaning F/G	2.52	3.15	3.07	0.19	0.25
Post - weaning F/G	2.35^{b}	2.63^{b}	3.18^{a}	0.12	0.01

Values in the same row (a, b) with different letters are significantly different ($P < 0.05$). LGM, in the form of chelates (lysine Mn: glutamic acid Mn = 1:1). MnSO4, in the form of sulfate Mn. SEM, standard error of means. Initial, d -14. Final, d 14. Overall, d -14 - d 14.
