# Supplementary material for: Effects of different manganese sources on nutrient digestibility, fecal bacterial community, and mineral excretion of weaning dairy calves
Source: Front Microbiol. 2023 May 18;14:1163468. doi: 10.3389/fmicb.2023.1163468 (PMC10232960; doi:10.3389/fmicb.2023.1163468)
Supplement: Supplementary file 3 [file Table_3.pdf]

Table3 Effects of different manganese sources on body size of calves (cm)

Item	CON	LGM	$MnSO_4$	SEM	*P*-value
Body height					
-14	86.40	84.70	84.80	0.54	0.38
-1	89.82	88.24	88.60	0.56	0.52
14	92.22	93.56	92.62	0.56	0.64
-14 - -1	3.42	3.54	3.80	0.37	0.92
-1 - 14	2.40	5.32	4.02	0.51	0.051
-14 -14	5.82	8.86	7.82	0.59	0.09
Body length					
-14	75.92	79.04	80.76	0.99	0.13
-1	82.22^{b}	89.14^{a}	90.78^{a}	1.35	0.01
14	91.74^{b}	96.22^{a}	98.54^{a}	1.07	0.02
-14 - -1	6.30	10.10	10.02	0.85	0.11
-1 - 14	9.52	7.08	7.76	0.82	0.49
-14 -14	15.82	17.18	17.78	1.01	0.75
Chest girth					
-14	93.32	97.74	99.50	1.14	0.06
-1	97.90^{b}	105.54^{a}	105.32^{a}	1.44	0.03
14	105.36^{b}	113.98^{a}	112.52^{a}	1.31	0.01
-14 - -1	4.58	7.80	5.82	0.65	0.12
-1 - 14	7.46	8.44	7.20	0.46	0.55
-14 -14	12.04^{b}	16.24^{a}	13.02^{b}	0.55	<0.01
Cannon circumference					
-14	10.50	11.00	10.62	0.27	0.76
-1	11.76	11.76	12.08	0.18	0.51
14	12.62^{b}	13.30^{ab}	13.76^{a}	0.18	0.02
-14 - -1	1.26	0.56	1.46	0.27	0.38
-1 - 14	0.86^{b}	1.74^{a}	1.68^{a}	0.17	0.05
-14 -14	2.12	2.30	3.14	0.29	0.34
Hip height					
-14	88.64	88.62	86.18	0.72	0.30
-1	92.36	92.22	90.14	0.66	0.33
14	94.52	96.44	93.98	0.64	0.28
-14 - -1	3.72	3.60	3.96	0.29	0.89
-1 - 14	2.16^{b}	4.22^{a}	3.84^{a}	0.31	<0.01
-14 -14	5.88^{b}	7.82^{a}	7.80^{a}	0.36	0.03
Hip width					
-14	16.18	16.74	16.80	0.25	0.57
-1	19.36	20.46	20.64	0.34	0.28
14	22.90	24.72	23.98	0.39	0.16
-14 - -1	3.18	3.72	3.84	0.25	0.56
-1 - 14	3.54	4.26	3.34	0.26	0.34
-14 -14	6.72	7.98	7.18	0.37	0.40

Values in the same row (a, b) with different letters are significantly different ($P < 0.05$). LGM, in the form of chelates (lysine Mn: glutamic acid Mn = 1:1). $MnSO_4$, in the form of sulfate Mn. SEM, standard error of means. -14, -1 and 14 represent calves at -14, -1 and 14 days after weaning, respectively.
