# Supplementary material for: Effects of different manganese sources on nutrient digestibility, fecal bacterial community, and mineral excretion of weaning dairy calves
Source: Front Microbiol. 2023 May 18;14:1163468. doi: 10.3389/fmicb.2023.1163468 (PMC10232960; doi:10.3389/fmicb.2023.1163468)
Supplement: Supplementary file 4 [file Table_4.pdf]

Table 4 Effects of different manganese sources on rectal temperature of calves (°C)

Item	Duration of treatment (days)						SEM	*P*-value		
	-14	-1	1	3	7	14		D	T	D×T
CON	38.54	38.64	38.68	38.68	38.54^{B}	38.56^{B}				
LGM	38.68	38.72	38.74	38.66	38.68^{B}	38.70^{B}	0.20	0.07	<0.01	0.01
$MnSO_4$	38.54^{b}	38.68^{b}	38.72^{b}	38.68^{b}	38.84^{Ab}	39.12^{Aa}				

Values in the same row (a, b) or in the same column (A, B) with different letters are significantly different ($P < 0.05$). LGM, in the form of chelates (lysine Mn: glutamic acid Mn = 1:1). MnSO4, in the form of sulfate Mn. SEM, standard error of means. D, effect of day. T, effect of group. D × T, interaction between day and group.
