# Supplementary material for: Effects of different manganese sources on nutrient digestibility, fecal bacterial community, and mineral excretion of weaning dairy calves
Source: Front Microbiol. 2023 May 18;14:1163468. doi: 10.3389/fmicb.2023.1163468 (PMC10232960; doi:10.3389/fmicb.2023.1163468)
Supplement: Supplementary file 5 [file Table_5.pdf]

Table 5 Effects of different manganese sources on fecal bacterial of weaning calves at phylum level.

Item	Group	Duration of treatment (days)					SEM	*P*-value		
		-1	1	3	7	14		D	T	D×T
Bacteroidota	CON	42.87	47.19	46.87	41.26	48.45	0.08	0.17	0.83	0.16
	LGM	45.10	46.13	42.79	41.37	45.86				
	$MnSO_4$	40.43	37.62	41.25	48.82	52.74				
Firmicutes	CON	49.71	47.16^{A}	48.17	56.25^{A}	49.28^{A}	8.13	0.15	< 0.01	0.20
	LGM	43.87	44.99^{A}	45.72	41.44^{B}	46.04^{AB}				
	$MnSO_4$	43.13	32.69^{B}	38.87	46.58^{B}	40.03^{B}				
Proteobacteria	CON	1.25^{B}	1.64	1.51	1.00	0.86^{B}	3.18	0.02	< 0.01	< 0.01
	LGM	2.49^{B}	3.21	3.69	3.62	3.72^{A}				
	$MnSO_4$	5.37^{Aa}	6.60^{a}	4.82^{a}	0.96^{b}	0.72^{Bb}				
Spirochaetota	CON	0.02^{B}	0.10^{B}	0.07^{B}	0.07	0.06	2.50	0.11	< 0.01	< 0.01
	LGM	6.34^{A}	1.94^{A}	6.39^{A}	2.36	2.17				
	$MnSO_4$	1.04^{B}	1.32^{A}	0.90^{B}	2.08	3.15				
Actinobacteriota	CON	1.46	1.96	1.53	1.00	1.22	1.59	0.54	0.48	0.77
	LGM	1.88	1.69	0.87	0.86	1.69				
	$MnSO_4$	1.52	1.45	2.64	0.99	2.93				
Verrucomicrobiota	CON	0.01^{B}	0.02	0.01	0.01	0.01	0.07	0.37	< 0.01	0.61
	LGM	0.11^{A}	0.13	0.05	0.02	0.09				
	$MnSO_4$	0.04^{B}	0.01	0.01	0.02	0.07				

Values in the same row (a, b) or in the same column (A, B) with different letters are significantly different ($P < 0.05$). LGM, in the form of chelates (lysine Mn: glutamic acid Mn = 1:1). MnSO4, in the form of sulfate Mn. SEM, standard error of means. D, effect of day. T, effect of group. D × T, interaction between day and group.
