# Supplementary material for: Effects of different manganese sources on nutrient digestibility, fecal bacterial community, and mineral excretion of weaning dairy calves
Source: Front Microbiol. 2023 May 18;14:1163468. doi: 10.3389/fmicb.2023.1163468 (PMC10232960; doi:10.3389/fmicb.2023.1163468)
Supplement: Supplementary file 6 [file Image_1.pdf]

Figure 1 Venn plots of the three groups within the whole period

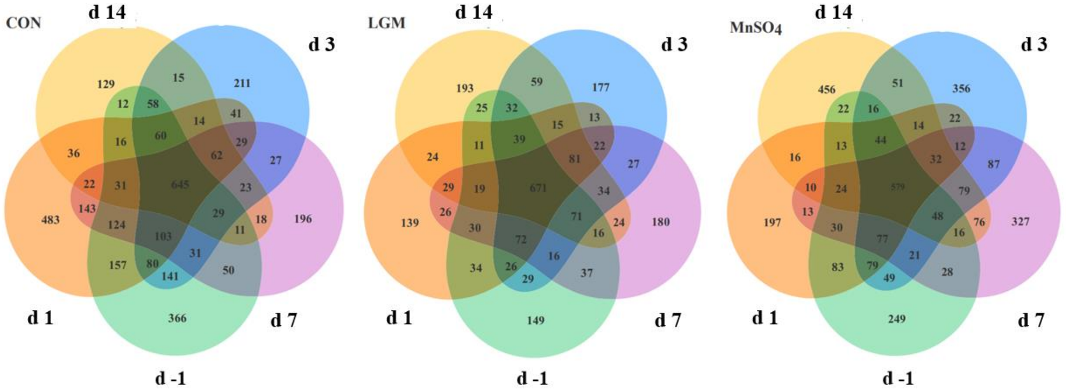

LGM, in the form of chelates (lysine Mn: glutamic acid Mn = 1:1). $MnSO_4$, in the form of sulfate Mn. d -1,d 1, d 3, d 7 and d 14 represent calves at -1, 1, 3, 7 and 14 days after weaning, respectively.
